# Age-Related Loss in Bone Mineral Density of Rats Fed Lifelong on a Fish Oil-Based Diet Is Avoided by Coenzyme Q_10_ Addition

**DOI:** 10.3390/nu9020176

**Published:** 2017-02-22

**Authors:** Alfonso Varela-López, Julio J. Ochoa, José M. Llamas-Elvira, Magdalena López-Frías, Elena Planells, MCarmen Ramirez-Tortosa, Cesar L. Ramirez-Tortosa, Francesca Giampieri, Maurizio Battino, José L. Quiles

**Affiliations:** 1 Institute of Nutrition and Food Technology “José Mataix Verdú”, Department of Physiology, Biomedical Research Center, University of Granada, 18100 Granada, Spain; alvarela@ugr.es (A.V.-L.); jjochoa@ugr.es (J.J.O.); maglopez@ugr.es (M.L.-F.); elenamp@ugr.es (E.P.); 2 Nuclear Medicine Service, Hospital Virgen de las Nieves, 18014 Granada, Spain; josem.llamas.sspa@juntadeandalucia.es; 3 Institute of Nutrition and Food Technology “José Mataix Verdú”, Department of Biochemistry and Molecular Biology II, Biomedical Research Center, University of Granada, 18100 Granada, Spain; mramirez@ugr.es; 4 Department of Pathology, Complejo Hospitalario de Jaén, 23007 Jaén, Spain; cesarl.ramirez.sspa@juntadeandalucia.es; 5 Department of Scienze Cliniche Specialistiche ed Odontostomatologiche, Università Politecnica delle Marche, 60131 Ancona, Italy; fgiampie@mta01.univpm.it (F.G.); m.a.battino@univpm.it (M.B.)

**Keywords:** antioxidants, dietary fat, *n*-3 PUFA, oxidative stress, ubiquinone

## Abstract

During aging, bone mass declines increasing osteoporosis and fracture risks. Oxidative stress has been related to this bone loss, making dietary compounds with antioxidant properties a promising weapon. Male Wistar rats were maintained for 6 or 24 months on diets with fish oil as unique fat source, supplemented or not with coenzyme Q_10_ (CoQ_10_), to evaluate the potential of adding this molecule to the *n*-3 polyunsaturated fatty acid (*n*-3 PUFA)-based diet for bone mineral density (BMD) preservation. BMD was evaluated in the femur. Serum osteocalcin, osteopontin, receptor activator of nuclear factor-κB ligand, ostroprotegerin, parathyroid hormone, urinary F_2_-isoprostanes, and lymphocytes DNA strand breaks were also measured. BMD was lower in aged rats fed a diet without CoQ_10_ respect than their younger counterparts, whereas older animals receiving CoQ_10_ showed the highest BMD. F_2_-isoprostanes and DNA strand breaks showed that oxidative stress was higher during aging. Supplementation with CoQ_10_ prevented oxidative damage to lipid and DNA, in young and old animals, respectively. Reduced oxidative stress associated to CoQ_10_ supplementation of this *n*-3 PUFA-rich diet might explain the higher BMD found in aged rats in this group of animals.

## 1. Introduction

Bone is continuously remodeled by two groups of cells with opposite activities, osteoblasts implicated in bone formation and osteoclasts implicated in bone resorption. Maturation processes and function of both kinds of cells can be altered during aging. When bone resorption rate exceeds osteoblasts rate of new bone formation by osteoclasts, calcium, collagen, and protein depletions will occur [1]. It is known that, regardless of sex, bone mass declines progressively with advancing age in humans [2]. Moreover, such decline is coupled with modifications of bone architecture and bone components organization [3]. All these age-associated changes [2,3,4] would explain the increased prevalence of osteoporosis reported in the elderly [5]. Osteoporosis has been defined as “a skeletal disorder characterized by compromised bone strength and predisposing to an increased risk of fracture” [6]. The former fact is particularly important since fractures are responsible for a significant mortality and morbidity in older people [7,8]. Osteoporosis is a disease that mostly affects elderly women, but it is also a major health problem for aged men [9], in whom this pathology shows an important association with substantial morbidity, mortality, and health care costs [10]. Because osteoporotic fractures involve considerable economic and healthcare burdens [11], promotion of effective prevention and treatment strategies to counterbalance them is imperative [12].

Amongst others, different nutritional factors have an important role in skeletal health during aging. Traditionally, calcium, vitamin D, and proteins have received special attention since they contribute to skeletal health by supporting bone matrix production and mineralization [13,14,15]. However, other dietary factors might influence bone and mineral homeostasis and could be important for long-term bone health [12]. In that sense, there is a growing body of evidence emphasizing the importance of dietary fat in bone aging and osteoporosis. Overall, high-fat diets, particularly those rich in saturated fats have been negatively associated with bone health [16,17], whereas polyunsaturated fatty acids (PUFA) seem to have some positive effects [18,19,20,21]. Notwithstanding, PUFA may also have important implications due to a wide range of possible mechanisms [22,23,24,25]. In relation to *n*-3 PUFA intake, there is evidence of a positive role of this type of fatty acid on bone mineral density (BMD), bone mineral content (BMC), or bone calcium levels [26,27,28,29,30,31,32,33,34,35]. This role of *n*-3 PUFA has been particularly notable when their effects are compared with those from *n*-6 PUFA in both humans [27] and animals [29,30,31,32,33,36]. However, some experiments with rats have suggested that this effect is dose-dependent [36] andthe positive role of *n*-3 PUFA could be conditioned by a ceiling [32,37]. In that sense, there are studies indicating some detrimental effects of a diet with a very high proportion of *n*-3 PUFA on other tissues and organs [38]. This is not entirely surprising since detrimental effects of diets rich in *n*-3 PUFA have been previously shown in other tissues [38]. Moreover, results from some in vitro studies have suggested possible negative effects on bone formation for some particular *n*-3 PUFA [39,40].

Along with dietary conditions and changes in several hormones levels, oxidative stress has also been shown to be implicated in the pathogenesis of osteoporosis or has been related to high bone loss during aging [41,42,43]. It is known that PUFA is highly susceptible to reactive oxygen species (ROS) attack, subsequently increasing the oxidative damage in the organism, especially during aging [44]. PUFA susceptibility to lipid peroxidation would also involve *n*-3 PUFA. In fact, it has been suggested that dietary compounds with antioxidant and anti-inflammatory properties could have promising bone protective effects [45,46]. Supplementation with antioxidants would preserve the advantages of PUFA on bone while preventing their deleterious effects and, in addition, modulate the effects of free radicals on bone health. In relation to aging, several studies in preclinical models have reported positive effects of dietary coenzyme Q (CoQ) [47,48,49]. CoQ is a key electron carrier in mitochondrial respiratory chain [6,23,26,27,28,29], and constitutes an important lipid-soluble antioxidant present in all biological membranes [50]. It has been shown to efficiently prevent oxidation of proteins, lipids, and DNA [51,52,53,54]. Likewise, other interesting physiological roles in cells related to these activities have been suggested for this molecule [50,55,56]. Based on these properties, the use of CoQ supplements under certain nutritional conditions associated with high oxidative stress levels could prove to be particularly interesting. In that sense, the effect of dietary CoQ has been investigated in the aging of some tissues and organs from rats fed with “pro-oxidant” PUFA-rich diets offering promising results [40,44,57,58,59,60,61]. However, knowledge about the effect of dietary CoQ on bone health is limited, and there are no studies on the effect of the combination of this antioxidant and *n*-3 PUFA-rich diet on bone health. On the other hand, research on diet and bone health have been mainly performed on postmenopausal rodent models (i.e., ovarectomized) using very young animals or directly on postmenopausal women with osteoporosis. However, rats reach skeletal maturity at the age of approximately 12 months [11], so results from many studies are not based on a good senile osteoporosis model. In addition, little research addressed male subjects. However, an age-dependent decline of bone mass and strength in sex steroid-sufficient female or male mice has been also reported [41]. In this context, the present study was designed to evaluate the potential of adding coenzyme Q_10_ (CoQ_10_) to a normocaloric and normolipidic diet with fish oil as a unique dietary fat source (rich in *n*-3 PUFA) for whole-life on bone aging and health in a male rat model of aging.

## 2. Materials and Methods

### 2.1. Experimental Design

Twenty-four male Wistar rats (*Rattus norvegicus*) weighing 80–90 g were housed three per cage and maintained in a 12-h light/12-h darkness cycle, with free access to food and water. The rats were randomly assigned into two experimental groups and fed from weaning until 24 months of age on a semi-synthetic and isoenergetic diet according to the AIN93 criteria [62] with the exception of dietary fat consisting only in fish oil. Diet composition was the following (in % weight/weight): 14% casein, 46.57% starch, 10% sucrose, 4% fish oil, 15.5% dextrose, 5% cellulose, 0.25% choline, 0.185 l-cystine, 1% vitamin mixture, and 3.5% mineral mixture. In addition, a supplemented version of each diet was prepared to reach 2.5 mg/kg per day of CoQ_10_. Two experimental groups were established: animals fed a fish oil-based diet without added CoQ_10_ (only the fish oil group) and those on the same diet but supplemented with CoQ_10_ (fish oil + CoQ). The rats were killed by cervical dislocation followed by decapitation, at the same time of the day, to avoid any circadian fluctuation. From each group, half of the rats were killed at 6 months of age and the other half at 24 months of age. Such ages were chosen because rats are respectively considered as young adults and elderly subjects, but not too old [63]. Blood was collected for plasma and serum isolation. Femur bones were isolated, physically cleaned from remaining soft tissues, weighted, and preserved in 70% ethanol until analysis. The animals were treated in accordance with the guidelines of the Spanish Society for Laboratory Animals, and the experiment was approved by the Ethical Committee of the University of Granada (permit number 20-CEA-2004).

### 2.2. Plasma Fatty Acid Profile and Total Coenzyme Q_10_ Levels Determination

Plasma fatty acid profile in rats was determined by following Lepage and Roy’s method [64] with previously described modifications [65]. Plasma levels of CoQ_10_ were assayed by high-performance liquid chromatography (HPLC) with electrochemical detection according to MacCrehan [66] as previously described [67]. A Spherisorb S5 ODS1 (Merck, Darmnstadt, Germany) column was used and ethanol/purified water 97:3 (*v/v*) acted as mobile phase. Retention times of individual standards were predetermined to identify CoQ_10_.

### 2.3. Determination of Bone Mineral Density

BMD and area were measured in the proximal half of the isolated femur bones by dual energy X-ray absorptiometry (DXA) using a Hologic QDR-4500 Elite densitometer (Hologic, Inc., Bedford, MA, USA). BMD was then calculated as bone mineral content (BMC)/area.

### 2.4. Determination of Bone Metabolism Markers, RANKL, Osteoprotegerin, and Hormones Circulating Levels

Serum levels of osteoprotegerin (OPG), receptor activator of nuclear factor kappa-B ligand (RANKL), osteocalcin, osteopontin, parathyroid hormone (PTH), and adenocorticotropin (ACTH) were measured simultaneously using a high sensitivity human cytokine multiplex immunoassay (Milliplex™ MAP, Merck Millipore, Billerica, MA, USA) according to the kit manufacturer’s instructions. Kits contained biotinylated antibodies, phycoerythrin-conjugated streptavidin, and different types of antibody-coated microspheres (Mixed Beads) with a specific antibody for each analyzed molecule. First, serum samples (25 µL) were incubated overnight at 4 °C with 25 µL of Mixed Beads. Then, the microsphere–protein complexes formed were washed and incubated at room temperature for 1 h with 50 µL of biotinylated antibodies, which specifically bind to proteins present on the microspheres. Finally, a final incubation with 50 µL of phycoerythrin-labeled streptavidin was carried out at room temperature for 30 min. After phycoerythrin-labeled streptavidin bind the biotinylated antibodies, the microspheres were loaded into a Luminex^®^ X-MAP Bio-Plex 200 System Bioanalyzer (Luminex Corp., Austin, TX, USA) where the assays were run. The bioanalyzer quantifies the amount of phycoerythrin fluorescence present in each of the distinct microsphere groups. Assays were calibrated using duplicate 8-point standard curves and at least 50 individual microspheres were counted for each analyte using the median fluorescence intensity for subsequent calculations. Machine performance was verified using quality control samples at low, medium, and high levels for each analyte. All standard and quality control samples were analyzed in a complex matrix to match the sample background. Serum samples were analyzed at optimized dilutions. RANKL/OPG ratio was calculated for each subject using the values of RANKL and OPG levels obtained by this method.

### 2.5. Urinary F_2_-Isoprostanes Determination

Urine was stored at −80 °C until analyzed for F_2_-isoprostanes. Total F_2_-isoprostanes were measured by a competitive enzyme immunoassay (R&D Systems, Minneapolis, MN, USA). Results were normalized to urinary creatinine.

### 2.6. Determination of DNA Strand Breaks

Single cell gel electrophoresis assay or comet assay was used to measure DNA strand breaks in peripheral blood lymphocytes [68]. For this, collected fresh blood was centrifuged. Then the “buffy coat” was isolated and diluted 1:1 with RPMI-1640 medium, layered onto an equivalent volume of Histopaque^®^-1077 (Sigma-Aldrich, St. Louis, MO, USA) to obtain peripheral blood lymphocytes. The assay was then carried out as previously described [57,69]. Briefly, isolated lymphocytes were suspended in a warmed 1% low melting point agarose in Phosphate-buffered saline (PBS) (pH 7.4) and pipetted onto microscope slides precoated with a (1% in PBS) high melting point agarose layer. Slides were maintained at 4 °C for 5 min and immersed in a lysis solution (2.5 M NaCl, 100 mM ethylenediaminetetraacetic acid (EDTA), 10 mM Tris at pH, 10, 1% Triton X-100 *v/v*) at 4 °C for 1 h. After lysis treatment, they were placed in an electrophoresis tank containing 0.3 M NaOH and 1 mM EDTA, pH 10 at 4 °C for 40 min to allow the separation of the two DNA strands. Finally, electrophoresis was performed at 1 V/cm and 300 mA for 30 min, and slides were washed three times for 5 min each with a neutralizing solution (0.4 M Tris, pH 7.5) at 4 °C before staining with 1 mg/mL of 4,6-diamidino-2-phenylindole (DAPI). Individual DAPI-stained nucleoids in each gel were examined under a UV-microscope Leica DM/LS (Leica Microsystems, Wetzlar, Germany) with an excitation filter of 435 nm and a magnification of 400 registered using a CCD camera (Hitachi, Tokyo, Japan) and analyzed with image analysis sofware Komet 5.0 (Kinetic Imaging Ltd., Liverpool, UK). A total of 100 comets per gel were count. The mean percent of DNA in the tail per gel was taken as a measure of DNA break frequency for each sample.

### 2.7. Statistical Analysis

Results were presented as mean and standard error of mean of six animals. Prior to any statistical analysis, all variables were checked for normal distribution and homogeneity of variances using the Shapiro–Wilk and the Levene tests, respectively. When a variable was found to follow a normal distribution, a Student’s *t*-test was used to compare different dietary groups at the same age or different age groups fed a same diet. Otherwise, the non-parametric Mann–Whitney *U*-test was used for the variables that did not follow a normal distribution (Urinary F_2_-Isoprostanes, DNA strand breaks and RANKL and ACTH levels). Data were analyzed using the IBM SPSS Statistics for Windows Version 22.0 (IBM Corp., Armonk, NY, USA). A *p*-value under 0.05 was considered significant in all cases.

## 3. Results

### 3.1. Animal Weights

During the 24-month follow-up, the fish oil group presented no significant weight differences (604.0 ± 23.3 g) compared to the fish oil + CoQ group (624 ± 20 g). From the observation of body weight evolution and food spillage, no differences concerning food intake were inferred between groups or in relation to age.

### 3.2. Plasma Fatty Acid Profile and Total Coenzyme Q_10_ Levels

**Figure 1:**
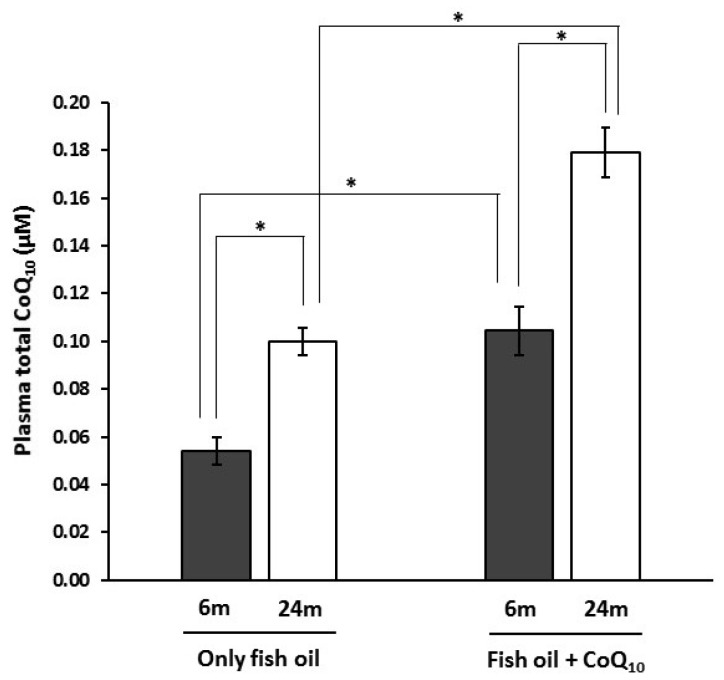
Effects of supplementation with coenzyme Q_10_ (CoQ_10_) on plasma total CoQ_10_ levels in 6- and 24-month-old (m) rats fed fish oil as dietary fat. Results are expressed as mean ± standard error of mean of six animals. * Statistically significant differences (*p* < 0.05) determined by the Student’s *t*-test.

The sum of plasma monounsaturated fatty acids (MUFA) in 6-month-old animals was 31.6 ± 2.4 g/100 g of total fatty acids. Concerning *n*-6 PUFA, a total value of 9.4 ± 0.4 g/100 g was found. For *n*-3 PUFA, 18.3 ± 1.2 g/100 g were noted. Such differences were maintained for old animals that showed values of 30.6 ± 2.1, 8.9 ± 1.1, and 19.5 ± 1.5 g/100 g for MUFA, *n*-6 PUFA, and *n*-3 PUFA, respectively; no differences with animals receiving CoQ_10_-supplementation were observed (data not shown). Concerning plasma CoQ_10_ content, those animals supplemented with CoQ_10_ for 6 months showed significantly higher concentrations than their non-supplemented counterparts (Figure 1). At 24 months of age, these differences were maintained.

### 3.3. Bone Mineral Density

**Figure 2:**
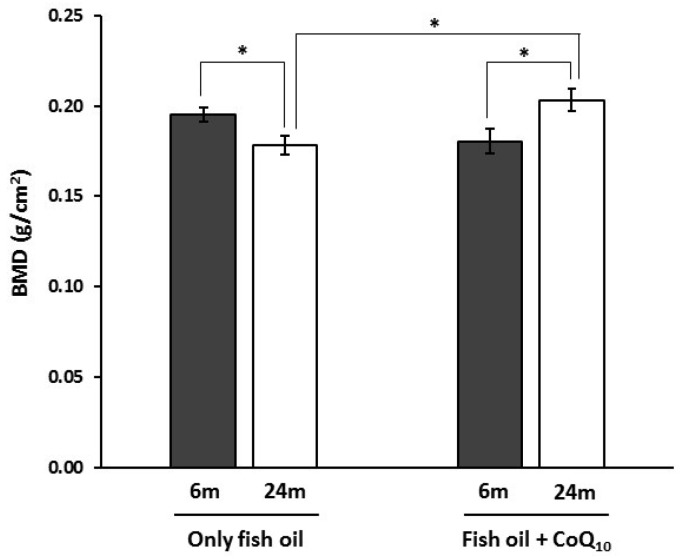
Effects of supplementation with coenzyme Q_10_ (CoQ_10_) on bone mineral density (BMD) in 6- and 24-month-old (m) rats fed fish oil as dietary fat. Results are expressed as mean ± standard error of mean of six animals. * Statistically significant differences (*p* < 0.05) determined by the Student’s *t*-test.

Results from BMD measurement are represented in Figure 2. Concerning aging, BMD was lower in old animals fed non-supplemented fish oil compared to their younger counterparts. However, in animals receiving CoQ_10_, older subjects displayed higher values. Regarding differences between dietary groups, BMD was lower in animals fed fish oil without CoQ_10_ but only at 24 months of age.

### 3.4. Urinary F_2_-Isoprostanes and DNA Strand Breaks

**Figure 3:**
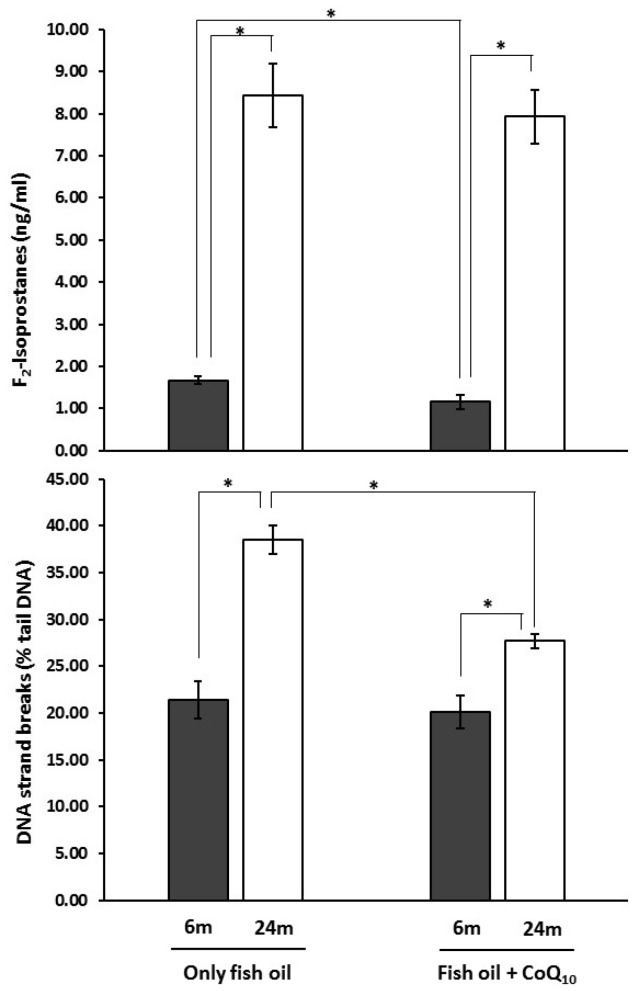
Effects of supplementation with coenzyme Q_10_ (CoQ_10_) on urinary levels of F_2_-isprostanes and DNA strand breaks in lymphocytes in 6- and 24-month-old (m) rats fed fish oil as dietary fat. Results are expressed as mean ± standard error of mean of six animals. * Statistically significant differences (*p* < 0.05) determined by the Mann–Whitney *U*-test.

Results from analysis of urinary F_2_-isoprostanes levels and DNA strand breaks in lymphocytes are shown in Figure 3. Concerning the CoQ_10_ effect, a lower value was found in CoQ_10_-supplemented animals at 6 months for F_2_-isoprostanes, but there were no differences at 24 months. In contrast, no differences were observed for DNA strand break at 6 months but animals receiving CoQ showed lower values at 24 months. Moreover, 24-month-old rats showed higher values than their younger counterparts for both in all cases.

### 3.5. Circulating Levels of Bone Metabolism Markers, OPG, RANKL, and Serum RANKL/OPG Ratio

**Figure 4:**
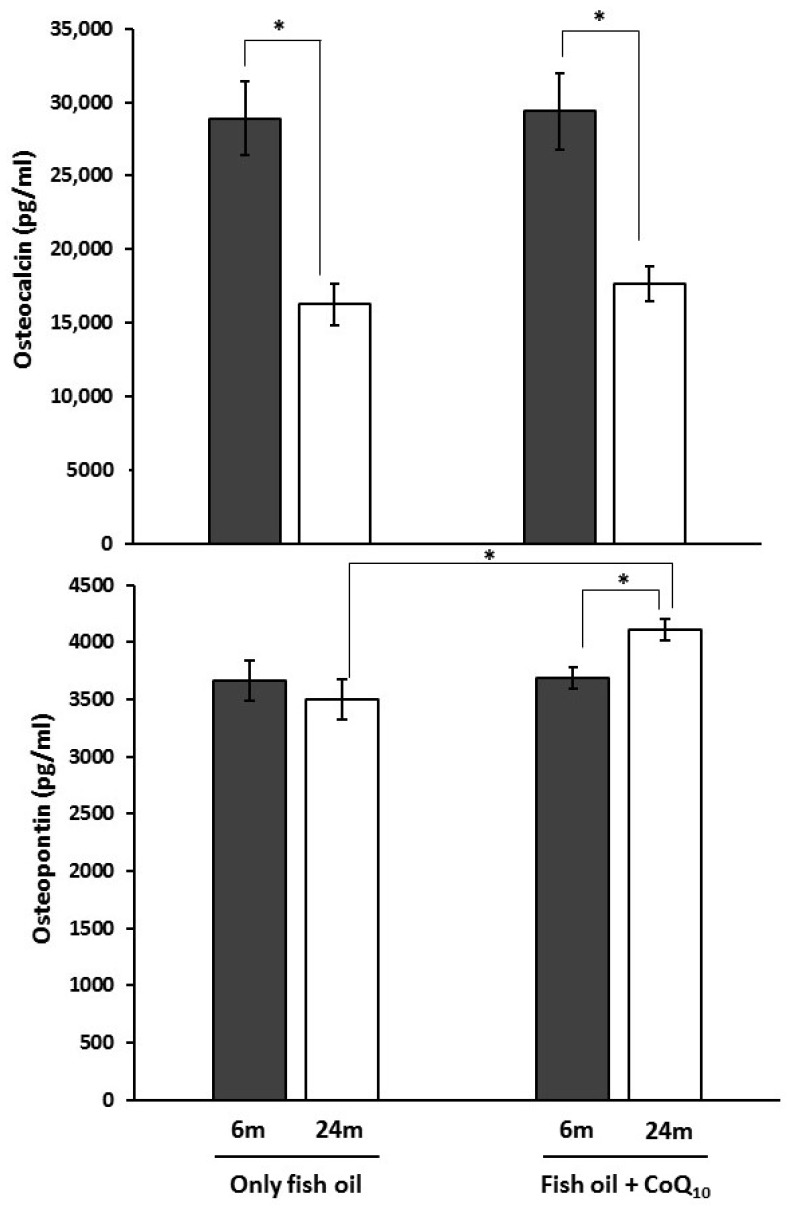
Effects of supplementation with coenzyme Q_10_ (CoQ_10_) on serum levels of bone metabolism markers (osteocalcin and osteopontin) in 6- and 24-month-old (m) rats fed fish oil as dietary fat. Results are expressed as mean ± standard error of mean of six animals. * Statistically significant differences (*p* < 0.05) determined by the Student’s *t*-test.

Results from analysis of serum levels of osteocalcin and osteopontin are presented in Figure 4. No differences were found between dietary groups for osteocalcin levels in serum at any age, but in the case of osteopontin, a higher level was found in older rats on non-supplemented diets compared to those receiving CoQ_10_ supplements at the same age. Older animals showed lower levels of osteocalcin than their younger counterparts. However, concerning osteopontin, only supplemented animals showed significant differences between age groups with 24-month-old animals displaying the highest values. 

Results from RANKL, OPG serum levels, and serum RANKL/OPG ratio are presented in Table 1. No statistically differences were found between animals kept on different diets at the same age for any of the mentioned parameters. Regarding aging, lower values were found for older animals in both groups for all.

### 3.6. Circulating Levels of PTH and ACTH

**Figure 5:**
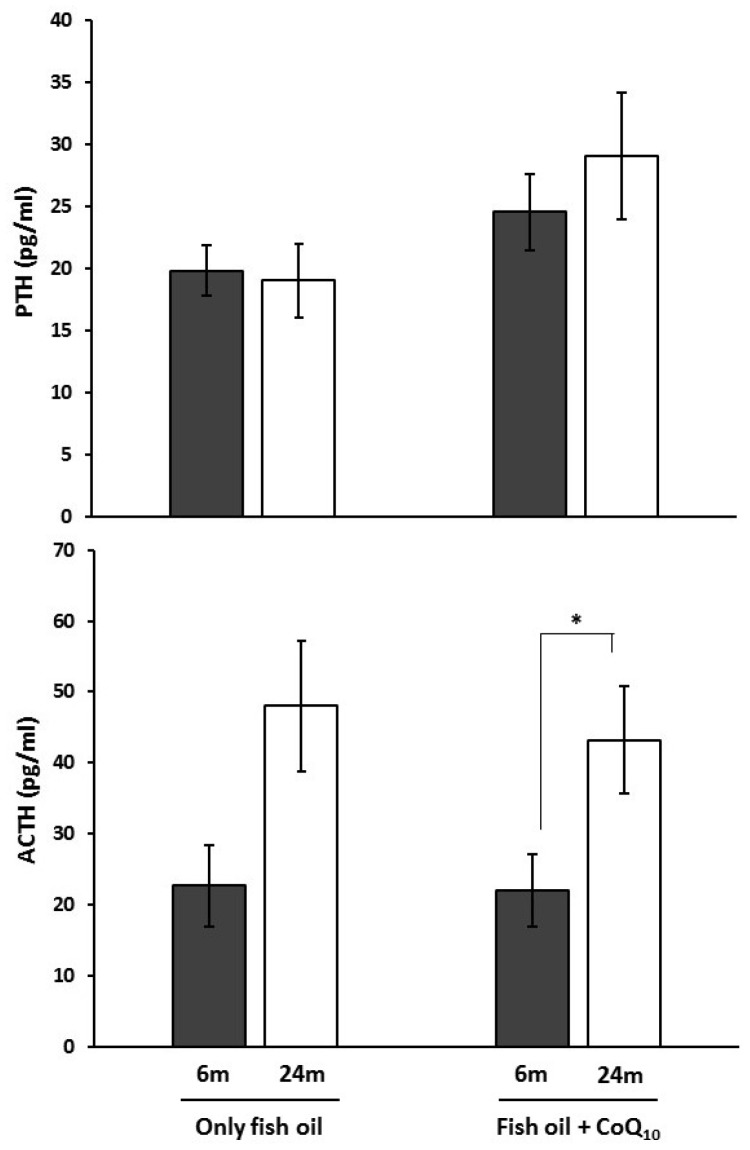
Effects of supplementation with coenzyme Q_10_ (CoQ_10_) on circulating levels of parathyroid hormone and adrenocorticotropin (ACTH) in 6- and 24-month-old (m) rats fed fish oil as dietary fat. Results are expressed as mean ± standard error of mean of six animals. * Statistically significant differences (*p* < 0.05) determined by the Mann–Whitney *U*-test.

Figure 5 shows collected data about the circulating level of analyzed hormones, PTH, and ACTH. Statistical analysis revealed no significant differences between dietary groups. Regarding age, a higher level was found in older animals but only when they received CoQ_10_ supplements.

## 4. Discussion

Different reports have indicated a positive association between aging and bone loss or risk of osteoporosis with increased probabilities of sustaining fractures [3,4,6]. Amongst other nutritional factors, dietary fat type has been shown to have implications in bone health. In particular, studies on *n*-3 PUFA effects have shown contradictory results that could be explained by the high susceptibility to lipid peroxidation associated to the different PUFA. With this background, here, rats were life-long fed *n*-3 PUFA-rich diets (with fish oil as unique dietary fat source) with or without addition of CoQ_10_, to test if this antioxidant and electron carrier can impose benefits of *n*-3 PUFA on BMD at different ages. BMD is a major parameter determining bone strength that is used for the diagnosis of osteoporosis and is expected to decrease during aging. Firstly, to attribute any effect on health to the dietary treatments, it was necessary to test if the diets provided were able to modify the lipid composition of body fluids and cellular structures. Results on plasma fatty acid profiles and CoQ_10_ plasma content confirmed a proper adaptation to the diets consumed since these resembled to those of the diets. This was expected according to previous studies on similar aging models [70,71].

Areal BMD is a major determinant of bone strength usually used to diagnose osteoporosis [72]. Results from the evaluation of BMD in rat proximal femur by DXA suggested an age-associated decrease in animals fed non-supplemented-diets, but when CoQ_10_ was added, the inverse change was observed, leading to higher values of BMD in old animals compared with their non-supplemented counterparts. Generally, to diagnose osteoporosis in humans, individual BMD values are expressed in relation to a reference population in standard deviation units. This allows reducing the difficulties associated with calibration of the instruments. In particular, when these units are used in relation to the young healthy population, this measurement is referred to as the *T*-score. Here, if values of young animals belonging to the fish oil group are used as reference to determine *T*-score, old animals receiving only fish oil would be in the low bone mass (osteopenia) category according to the WHO proposal modified by the International Osteoporosis Foundation for men and women [73,74], whereas BMD in fish oil + CoQ group at 24 months was normal. However, the clinical significance of these categories lies in the fracture risks that arise. A strong gradient of risk has been reported for fracture prediction using DXA, particularly at the proximal femur and lumbar spine. Namely, it was indicated that the risk of hip fracture increased 2.6-fold for each SD decrease in BMD at the femoral neck [75]. In this sense, it has been estimated that women in the threshold for osteopenia have a 50% lifetime risk of fracture [76].

Therefore, life-long supplementation with CoQ_10_ has an interesting potential for the prevention of osteoporosis and the maintenance of bone strength. The positive effect of CoQ on BMD has also been suggested by a study in rats where short-term treatment with CoQ_10_ prevented bone and mineral content loss induced by spinal cord injury [77]. According to Jacob and Nair [78] and the U.S. Food and Drug Administration (FDA) proposal for the Human Equivalent Dose (HED), the dose administrated to rats from the present study would be 0.26 mg/kg per day if we assume a weight of 70 kg as male mean value. This implies that a 70 kg man would need to take at least 18.43 mg to note similar effects. In previous interventions with CoQ in humans, doses ranged from 100 to 2400 mg per day [55]. This difference is very important, because although most human studies gave higher doses for limited periods of time when people were older or had a health problem, we verified that maintaining CoQ throughout life at lower doses has an interesting preventive role. In the present study, CoQ_10_ in its oxidized form (ubiquinone) was used, instead of ubiquinol, given its easy commercial access together with its better stability, as well as lower costs. However, ubiquinol is also available on the market, but at a higher price, and it has been shown to be more efficiently absorbed [79,80,81]. Therefore, ubiquinol might be an interesting alternative to the use of ubiquinone, particularly for short-time periods or at lower doses.

Along with BMD, bone remodeling is also a major determinant of bone strength since it influences bone structure. In this sense, studies reporting the positive effects of *n*-3 PUFA or fish oil on bone mass or density have shown increases [82], but also decreases in markers of bone formation [83]. On the other hand, there are no data about the role of CoQ on this issue. In the present study, serum levels of osteocalcin and osteopontin were measured to assess changes in bone turnover and resorption. Osteocalcin was lower for the aged groups, irrespective of the treatment. On the other hand, only an age-associated increase in rats receiving CoQ was observed in relation to osteopontin. Osteopontin is a protein from bone extracellular matrix where it controls mineralization, coupling of bone formation, and attachment of osteogenic cells to the bone matrix and resorption [84,85]. However, this protein is also expressed in a variety of tissues different from bone where it is usually expressed only in response to stimulus such as inflammation [86]. These include bone marrow derived gland cells, cartilage, dentine, cementum, kidney, brain, vascular tissues, specialized epithelia found in mammary, salivary, sweat glands, in bile and pancreatic ducts, in distal renal tubules, gut, and activated macrophages and lymphocytes [87]. Likewise, many physiological roles such as calcification, immune system modulation, inflammation, regulation of cell adhesion migration, and cell survival have been attributed to osteopontin [88]. Different evidence suggests that it participates in multiple physiological and pathological processes including pathological calcifications of soft tissues, wound healing, cardiovascular diseases and kidney diseases [87]. In fact, its plasma levels have been found to be associated with various inflammatory diseases, including cardiovascular diseases in clinical studies [86]. Consequently, changes in its blood levels observed here could reflect some physiological changes in other parts of the body and not necessarily the bone resorption rate. On the other hand, osteocalcin is an extracellular matrix protein that is released into blood during both new matrix formation and existing matrix breakdown [89]. Thus, its serum levels can be indicative of the rate of bone turnover. However, according to these results, the CoQ effect was not relevant to it.

As has been stated, bone health, in particular BMD, has been associated with oxidative stress levels in humans [90,91,92,93,94]. Moreover, in male and female mice, an association with age-dependent decline of bone mass and strength in sex steroid-sufficient animals has been also reported [41]. Thus, the effect of dietary CoQ_10_ on bone found here could be directly or indirectly related to its antioxidant activity. In that sense, it has been previously reported that the addition of this molecule to other PUFA-rich diets, namely *n*-6 PUFA, attenuated or reduced oxidative stress during aging [57,58,59,61]. To test the possible consequences on oxidative stress of supplementation with CoQ in animals from the present study (all of them fed an *n*-3 PUFA-rich diet), two markers of oxidative damage were used, each for a different type of macromolecules. These were urinary F_2_-isoprostanes and DNA strand breaks in lymphocytes estimated by a single cell gel electrophoresis assay (comet assay). These markers could provide a good picture about oxidation in lipids and DNA, respectively. Differences found between age groups for both markers suggest that aged animals had higher oxidative stress. Supplementation with CoQ_10_ prevented accumulation of DNA damage with aging, in parallel to the prevention of BMD decrease. This could contribute to the lowest loss of BMD found in CoQ_10_-supplemented animals. Supporting the positive effect of CoQ_10_ on DNA oxidative damage, it has been shown that CoQ_10_ addition to Mediterranean diet improved DNA repair systems in humans [95,96], which may also help by preventing DNA damage accumulation. Concerning lipids, oxidative damage was lower in young animals when these received supplementation but not in older subjects. This could indicate that, under conditions of the present study, dietary CoQ reduced oxidative stress from early in life, but this was not enough to lead to differences in lipid damage among older animals. However, it would prevent accumulation of damage to DNA, which would appear later in life.

As is well known, several hormones can influence bone metabolism [97,98]. Here, PTH and ACTH were evaluated. PTH is a hormone with a dual role in relation bone mass. It is known that this hormone induces bone resorption and mobilizes bone calcium into blood to balance low calcium levels in blood. Thus, high levels of PTH have been associated with a higher bone resorption rate. However, it has been reported that moderate levels of PTH maintained during short periods of time could even increase bone mass despite punctual increases in resorption rate [99]. Amongst other mechanisms, it has been previously suggested that *n*-3 PUFA’s protective effect on aging-induced loss of BMD was due to a modulation of systemic calcitrophic hormones, including PTH, in a study in male rats [83]. In the present study, no relevant effects of dietary CoQ_10_ were observed on PTH. Concerning ACTH, it has been traditionally considered to produce a negative impact on bone mass as a consequence of its activity stimulating cortisol release [100,101]. However, it has been proposed that cortisol might not have a detrimental effect on bone proliferation or differentiation at physiological levels [102]. An ACTH receptor, MC2R, is expressed in osteoblast and inflammatory cells [101,103]. In vitro experiments have shown anabolic effects for ACTH in osteoblasts at high concentrations, whereas lower concentrations opposed osteoblast differentiation [97]. In addition, MC2R expression is strongest at sites of active bone deposition, and thus ACTH response probably varies with osteoblastic activity or stage of osteoblast differentiation. In relation to CoQ_10_, it has been shown that pituitary-dependent adrenal diseases with defective activity of pituitary-adrenal axis are associated with low levels of this molecule [104,105]. Here, ACTH serum levels were significantly higher in old animals fed CoQ_10_ compared with their younger counterparts, but this difference was not significant in animals not receiving CoQ_10_. However, no significant effect was found for CoQ_10_, so no clear conclusion can be inferred from the study of these markers. Notwithstanding, isolated osteoclasts synthesize and release ACTH into the culture medium [106], so maybe local concentrations are more important for stimulation of bone formation.

Furthermore, a possible implication of osteoclastogenesis regulation in CoQ_10_ effect on BMD was also evaluated in the present study by serum RANKL/OPG ratio. RANKL and OPG are two regulatory proteins with antagonist roles. RANKL is a membrane protein presented by osteoblasts and other bone cells, which binds to the RANK receptor on osteoclast precursor cells, activating NF-κβ and promoting osteoclast differentiation [106]. OPG is a soluble decoy receptor for RANKL that competitively antagonizes RANKL–RANK interaction, inhibiting osteoclastogenesis [107]. Therefore, the RANKL/OPG ratio would indicate bone resorption rate [108]. Results from the present study suggest that the serum RANKL/OPG ratio is lower with aging in all groups, which is in agreement with previous studies [109,110]. Nevertheless, in the present study, there is not any statistically significant effect of CoQ_10_ on the RANKL/OPG ratio for any of the dietary groups. Likewise, when RANKL and OPG levels are observed separately, a similar pattern was observed. This difference associated to age is consistent with results from other studies in similar models of aging [61,111]. Since these diets did not lead to age-associated increases in RANKL, oxidative stress level could influence osteoclasts response to RANKL stimulation. In fact, it has been suggested that intracellular ROS can act as “secondary messengers” in osteoclasts or osteoclast progenitor cells enhancing their differentiation [112,113]. In particular, the RANKL pathway downstream transcription factor NF-κB is also an oxidative stress-responsive and may be activated by free radicals. Therefore, the higher oxidative stress levels observed in aged rats particularly when they were on diets rich in polyunsaturated fat [44,60,68,70,114,115,116] could led to an increase in sensitivity to RANKL-stimulation, explaining, at least in part, the existence of an age-associated increase in osteoclastogenesis and bone resorption despite the decrease in RANKL levels. Thus, the in vivo antioxidant activity shown by CoQ_10_ in the present study during aging might help to prevent bone loss as a consequence of a reduction of ROS in osteoclasts and osteoclast progenitor cells. In vitro studies support this hypothesis. In fact, in RANKL-stimulated bone marrow-derived monocytes and RAW 264.7 cells, a model of osteoclast, CoQ_10_ inhibited osteoclastogenesis [112,113]. Evidence suggests that CoQ_10_ strongly suppressed H_2_O_2_-induced IκBα, p38 signaling pathways [110]. Therefore, and considering results from the present study, oxidative stress might help to explain age-associated changes in BMD, as well as the preservation of BMD found in old animals supplemented on CoQ_10_. Still, oxidative stress can also damage other cells implicated in bone metabolism such as osteoblasts as well as suppress their differentiation [117].

## 5. Conclusions

Compared with young subjects, a lower BMD was found in old animals maintained on an *n*-3 PUFA-rich diet based on fish oil. This lower BMD associated to age was prevented by CoQ_10_ supplementation that even improved this parameter compared to young animals. In parallel, CoQ_10_ supplementation led to the prevention of oxidative damage to lipids and to DNA in young and old animals, respectively. However, CoQ_10_ had no clear effect in other factors affecting bone metabolism. Altogether, results from the present study suggest that, under our experimental conditions, oxidative stress might be among the causes of the lower BMD observed during aging. For this reason, it is feasible that CoQ_10_ could collaborate in the reduction of age-related loss of bone mass due to its antioxidant activity. According to other studies, this could be due, at least in part, to mitigation of osteoclastogenesis or osteoclast activity as consequence of intracellular ROS scavenging. Nevertheless, although such studies suggest that the dietary CoQ_10_ effect on age-related changes in BMD is mediated by a decrease in resorption rate, this molecule could also influence bone formation but the effect of this molecule, or oxidative stress, on osteoblasts has not been widely studied. Therefore, to clarify this issue, markers for each cell type formation and activity such as collagen breakdown products, TRAP5b, should be performed in the future. In the same way, it is known that many osteogenic progenitor cells turn out to be adipocytes during aging [118]. Since this phenomenon also reduces bone acquisition capacity, it will be interesting also to evaluate the possible effect of dietary fat and antioxidants on it. Therefore, these and other possible mechanisms for explaining CoQ_10_ effects on age-related BMD preservation need to be further investigated in this type of aging model. Likewise, it is imperative to extend this research to female individuals, although a more complex experimental design which would fit better to what happens in humans would be required.

## Figures and Tables

**Table 1:** Effects of supplementation with coenzyme Q_10_ (CoQ_10_) on serum levels of osteoprotegerin (OPG), receptor activator of the nuclear factor κB ligand (RANKL), and RANKL/OPG ratio in 6- and 24-month-old (m) rats fed fish oil as dietary fat.

Diet	Only Fish Oil		Fish Oil + CoQ_10_	
Age	6 Months	24 Months	6 Months	24 Months
RANKL (pg/mL)	46.97 ± 5.74 *	30.20 ± 3.99	50.76 ± 2.97 ^#^	28.59 ± 5.18
OPG (pg/mL)	751.87 ± 76.64 *	1161.08 ± 108.56	708.89 ± 82.98 ^#^	1177.95 ± 73.92
RANKL/OPG	0.067 ± 0.011 *	0.028 ± 0.005	0.075 ± 0.007 ^#^	0.024 ± 0.005

Results are expressed as mean ± standard error of mean of six animals. * Statically significant differences (*p* < 0.05) between 6 and 24 months old animals fed non-supplemented fish oil; ^#^ Statically significant differences (*p* < 0.05) between 6 and 24 months old animals fed CoQ_10_-supplemented fish oil.
